# Born this way: Hippocampal neurogenesis across the lifespan

**DOI:** 10.1111/acel.13007

**Published:** 2019-07-12

**Authors:** Danka A. Kozareva, John F. Cryan, Yvonne M. Nolan

**Affiliations:** ^1^ Department of Anatomy & Neuroscience University College Cork Cork Ireland; ^2^ APC Microbiome Ireland University College Cork Cork Ireland

**Keywords:** adolescence, aging, hippocampus, lifespan, memory, mood, neurogenesis

## Abstract

The capability of the mammalian brain to generate new neurons through the lifespan has gained much attention for the promise of new therapeutic possibilities especially for the aging brain. One of the brain regions that maintains a neurogenesis‐permissive environment is the dentate gyrus of the hippocampus. Here, new neurons are generated from a pool of multipotent neural progenitor cells to become fully functional neurons that are integrated into the brain circuitry. A growing body of evidence points to the fact that neurogenesis in the adult hippocampus is necessary for certain memory processes, and in mood regulation, while alterations in hippocampal neurogenesis have been associated with a myriad of neurological and psychiatric disorders. More recently, evidence has come to light that new neurons may differ in their vulnerability to environmental and disease‐related influences depending on the time during the life course at which they are exposed. Thus, it has been the topic of intense research in recent years. In this review, we will discuss the complex process and associated functional relevance of hippocampal neurogenesis during the embryonic/postnatal period and in adulthood. We consider the implications of hippocampal neurogenesis during the developmentally critical periods of adolescence and older age. We will further consider the literature surrounding hippocampal neurogenesis and its functional role during these critical periods with a view to providing insight into the potential of harnessing neurogenesis for health and therapeutic benefit.

## 1 INTRODUCTION

The process of generating functional neurons from stem and precursor cells in the central nervous system (CNS) was originally believed to occur strictly during embryonic and early postnatal development in mammals. A century ago, this dogma was challenged with the discovery of neurogenesis in the adult brain. Ezra Allen was the first to demonstrate that mitosis persisted in the lateral walls of adult albino rats (Allen, 1918). Several decades later, Altman and Das followed up this research and determined that neurogenesis occurred in the adult rat and guinea pig hippocampus (Altman & Das, 1965, 1967). However, it was not until the 1990s that the concept of functional hippocampal neurogenesis began to emerge (Palmer, Ray, & Gage, 1995; Palmer, Takahashi, & Gage, 1997; Suhonen, Peterson, Ray, & Gage, 1996). Since then, evidence has accumulated to demonstrate the existence of this process in the human hippocampus throughout the lifespan (Boldrini et al., 2018; Eriksson et al., 1998; Moreno‐Jiménez et al., 2019; Spalding et al., 2013). While controversies still persist, it is generally accepted that neurogenesis occurs in the adult hippocampus and has functional relevance (Kempermann et al., 2018).

Animal studies have provided substantial evidence that newly born granule cells in the adult hippocampus are electrophysiologically functional and become integrated in existing neuronal networks (van Praag et al., 2002; Toni & Schinder, 2015). Behavioural studies suggest that these new granule cells play important roles in certain types of cognitive processing such as spatial learning and memory, and in mood regulation (Balu & Lucki, 2009; Bond, Ming, & Song, 2015; Zhao, Deng, & Gage, 2008). Moreover, impaired hippocampal neurogenesis has been reported in neurodegenerative and psychiatric conditions (Balu & Lucki, 2009) and efforts to develop therapeutic strategies that employ the hippocampal neural stem cells (NSCs) are ongoing. It is becoming apparent that neurogenic processes or rates may differ at various times during the life course. New neurons may thus differ in their response to environmental influences and disease‐modifying factors at various times during life, which also has functional implications. Thus, hippocampal neurogenesis and its functional relevance have been the topic of intense research during the last decades. In this article, we review recent developments in understanding the similarities and differences in the origins and processes of embryonic and adult hippocampal neurogenesis. We consider the implications of hippocampal neurogenesis during the developmentally critical periods of adolescence and older age. We further evaluate the existing evidence regarding the functional roles of adult‐born granule cells during these critical periods with a view to providing insight into the potential of harnessing hippocampal neurogenesis for health and therapeutic benefit.

## 2 EMBRYONIC AND POSTNATAL HIPPOCAMPAL NEUROGENESIS: SIMILARITIES AND DIFFERENCES TO THE ORIGINS OF ADULT HIPPOCAMPAL NEUROGENESIS

In rodents, neurons begin to develop from neural epithelial cells (NECs), which are considered the earliest NSCs, at approximately embryonic day (E) 9‐9.5. By E15‐E17.5, all neurons comprising the cortical and subcortical areas have been generated and have migrated (Jin, 2016; Semple, Blomgren, Gimlin, Ferriero, & Noble‐Haeusslein, 2013). The dentate gyrus (DG) of the hippocampal formation is developed from a separate source of progenitor cells (the dentate neuroepithelium; DNE), which may have important consequences for the neurogenic permissive environment that emerges postnatally (Urbán & Guillemot, 2014). Hippocampal neurons are produced from the DNE from E13.5, and by E17.5, the hippocampal fissure is formed. The dentate precursor cells migrate and accumulate within the fissure to comprise the future layer of NSCs of the adult subgranular zone (SGZ) or to become neurons that form the granular cell layer (GCL; Urbán & Guillemot, 2014). With regard to the embryonic origin of NSCs evident in this hippocampal neurogenic niche in adulthood, it has been proposed that the NSCs in the SGZ come from the DNE itself at early stages of the embryonic period (Seki et al., 2014), while it has also been suggested that they are generated perinatally in the ventral DG (vDG), and subsequently migrate to the dorsal DG (dDG; Berg, Bond, Ming, & Song, 2018; Li, Fang, Fernández, & Pleasure, 2013). A recent report tracing the origin of rodent neural precursor cells (NPCs) has shed light on this question by showing that a common population of NSCs contributes to the DG neurogenesis throughout development and adulthood and that NSCs shift from quiescence to active state at different time points (Berg et al., 2019). They thus propose that adult hippocampal neurogenesis may represent a lifelong extension of development that maintains heightened plasticity. In support, it has been reported using a single‐cell RNA (ribonucleic acid) sequencing approach that adult neurogenesis and early postnatal development share highly similar transcriptional trajectories (Hochgerner, Zeisel, Lönnerberg, & Linnarsson, 2018).

While most of the granule cells of the rodent hippocampus are generated up until postnatal day (P)10 (Altman & Bayer, 1990; Piatti, Espósito, & Schinder, 2006), NSCs remain abundant in the developing brain until P14 (Malatesta, Hartfuss, & Götz, 2000) when they start differentiating into NPCs. This is followed by their transformation to neuroblasts and finally to mature excitatory granule cells that integrate in the circuitry by P21 (Kriegstein & Alvarez‐Buylla, 2009). A comprehensive analysis of NPCs in mice aged from P7 and P28 revealed that not only did the number of NPCs decrease over this developmental period, but also that the genetic profile of the NPCs from the two ages was markedly different implying early adulthood senescence (Gilley, Yang, & Kernie, 2011). Based on the recent reports by Hochgerner et al. (2018) and Berg et al. (2019), we argue that the different genetic profile of NPCs observed by Gilley et al. (2011) could be explained by distinct signals that activate quiescent NSCs depending on whether they are involved in embryonic or adult neurogenesis. Thus, both embryonic and adult NSCs can have common origin but generate NPCs with different genetic profile.

An important regulator of CNS development is microglia, the innate immune cells of the CNS. Microglia have a wide range of functions across the lifespan and across different regions of the CNS (reviewed by Boche, Perry, and Nicoll (2013)). To begin with, during CNS development microglia contribute to the formation of neuronal circuits and promote their survival through the release of neurotrophins, growth factors and cytokines (Deverman & Patterson, 2009; Nayak, Roth, & McGavern, 2014). Furthermore, microglia have been shown to prune redundant neurons by initiating cell death programmes followed by phagocytosis or to clear cellular debris after apoptosis. They have also been shown to promote NPC survival in the developing CNS, to engulf less active intact synapses and to regulate activity‐dependent synaptic remodelling (reviewed by Reemst, Noctor, Lucassen, & Hol, 2016; Schafer & Stevens, 2015). A unique phenotype of neonatal microglia has now been identified and is shown to be involved in signalling in order to facilitate myelination and neurogenesis in the developing brain (Wlodarczyk et al., 2017).

### 2.1 Function of hippocampal neurogenesis during embryonic and postnatal development

The function of neurogenesis during embryonic development is to populate the various regions of the CNS with different types of neurons derived from NSCs of the neural tube (Kandel, Schwartz, & Jessell, 2000; Table 1). The hippocampal formation is largely developed by E20 in rodents (Bayer, 1980) and by 20 weeks of gestation in humans (Gómez & Edgin, 2016). However, volumetric development persists to P21 in rodents and to 2 years of age in humans (Ainge & Langston, 2012; Hevner, 2016). Given that the postnatal period of the rodent is markedly different from that in humans, it was recently proposed that to objectively compare neurogenesis across species, neurogenic rates should be reflected as proportion over the lifespan of the species, rather than aligned to age postbirth (Snyder, 2019). In a recent study, tracing the survival of rat granule cells, it was demonstrated that neurons born during embryonic development (E19) and early adolescence (P21) survived throughout adulthood (2–6 months), while the cells generated at P6 displayed 15% cell death during adulthood. Thus, the authors proposed that early postnatal granule cells have a unique function in hippocampal plasticity (Ciric, Cahill, & Snyder, 2019). The function of early life postnatal hippocampal neurogenesis appears to be related to weakening existing memories and information storage in favour of strengthening the ability to learn new things and to acquire new information through rapid continuous generation of large number of new granule cells (Table 1; for extensive review, see Akers et al. (2014, Josselyn and Frankland (2012). The adaptive value of this function is considered to be in the rapid clearance of old information that may not be useful, in order to facilitate increased capacity and reduced interference between memories.

**Table 1:** Function of hippocampal neurogenesis through the lifespan

Intervention	Function of neurogenesis	Species	References
Embryonic & Early Postnatal development			
IHC, volumetric & morphologic analysis	Populate the hippocampal formation with neurons	Rodents (by E20) Humans (by gest. week 20)	Bayer (1980), Gómez and Edgin (2016)
	Complete volumetric development of the DG	Rodents (P21) Humans (2 years old)	Ainge and Langston (2012), Hevner (2016)
Inhibition/Enhancement of neurogenesis & behavioural interventions	Weakening existing memories and information storage in favour of strengthening the ability to learn new things and to acquire new information (infantile amnesia)	Shown across species	Akers et al. (2014), Josselyn and Frankland (2012)
Adolescence			
Behavioural interventions & inducing increase in neurogenesis (exercise)	Affiliative behaviour	Mice	Wei et al. (2011)
	Processing of stress‐inducing stimuli (social defeat; social isolation)	Mice	Kirshenbaum et al. (2014), Kozareva et al. (2018)
Inducing increase in neurogenesis (fluoxetine)	Response to antidepressant treatment of vDG newborn neurons	Rats	Klomp et al. (2014)
Ablation of neurogenesis through irradiation during early life/adolescence	Impaired fear conditioning and MWM performance in adulthood	Rats & Mice	Achanta et al. (2009), Rola et al. (2004)
	Impaired IQ scores and cognitive performance	Human	Rodgers et al. (2013)
Impaired neurogenesis	Implications in psychiatric disease	Rodents	Reviewed by Hueston et al. (2017)
Adulthood			
Ablation of neurogenesis through irradiation	Impaired fear conditioning but not spatial memory (MWM, Y‐maze)	Mice	Saxe et al. (2006)
	Impaired spatial learning & memory in Barnes maze but not MWM	Mice	Raber et al. (2004)
	Normal spatial learning and memory (MWM) and anxiety‐like behaviour (novelty suppressed feeding test)	Mice	Meshi et al. (2006)
	Impaired pattern separation (radial arm maze & touch screen—for similar but not distinct spatial locations)	Mice	Clelland et al. (2009)
	Blocked antidepressant‐induced enhanced behavioural performance and neurogenic levels	Mice	Santarelli et al. (2003)
	Impaired fear conditioning and place learning (T‐maze), but normal MWM and NOR	Rats	Madsen, Kristjansen, Bolwig, and Wörtwein (2003), Winocur, Wojtowicz, Sekeres, Snyder, and Wang (2006)
	Impaired long‐term spatial memory (MWM)	Rats	Snyder et al. (2005)
	Blocked pharmacologically induced enhanced behavioural performance and neurogenic levels	Rats	Jiang et al. (2005)
	Brain cancer treated with cranial radiation therapy associated with cognitive decline (impaired memory, attention and executive function)	Human	Greene‐Schloesser et al. (2013), Sarkissian (2005)
Pharmacological ablation of neurogenesis	Impaired ability to acquire trace memories, but not fear memories or perform in the MWM (spatial memory) & EPM (anxiety‐like behaviour)	Rats	Shors et al. (2001), Shors, Townsend, Zhao, Kozorovitskiy, and Gould (2002)
	Impaired memory in NOR	Rats	Bruel‐Jungerman, Laroche, and Rampon (2005)
Transgenic/knockdown methods for ablation (each study has targeted different genes)	Impaired spatial learning and memory (MWM), but normal fear conditioning	Mice	Zhang, Zou, He, Gage, and Evans (2008)
	Impaired pattern separation (radial maze for similar but not distinct spatial locations)	Mice	Clelland et al. (2009)
	Blocked antidepressant‐induced enhanced behavioural performance and neurogenic levels	Mice	Santarelli et al. (2003)
	Increased anxiety‐like behaviour (EPM)	Mice	Revest et al. (2009)
	Impaired spatial learning, but not memory (MWM)	Mice	Zhao et al. (2003)
	Impaired spatial memory consolidation, but not learning	Mice	Zhao et al. (2007)
	Impaired spatial learning and memory (MWM)	Mice	Shimazu et al. (2006)
	Impaired pattern separation (recognition memory for similar but not distinct locations)	Rats	Bekinschtein et al. (2014)
Enhancement of neurogenesis through learning and/or enrichment	Classic study illustrating that hippocampal‐dependent associative learning enhances (doubles) the number of adult‐born neurons in the hippocampal formation	Rats	Gould, Beylin, Tanapat, Reeves, and Shors (1999)
	Enhanced long‐term memory in NOR	Rats	Bruel‐Jungerman et al. (2005)
	Enhanced learning (MWM) and long‐term potentiation	Mice	van Praag, Christie, Sejnowski, and Gage (1999)
	Enhanced long‐term pattern separation (recognition memory for similar objects in NOR)	Mice	Bolz, Heigele, and Bischofberger (2015)
	Enhanced long‐term pattern separation (recognition memory for similar locations in NOL)	Mice	Creer, Romberg, Saksida, van Praag, and Bussey (2010)
Pharmacological enhancement of neurogenesis	Enhanced antidepressant effects on novelty suppressed feeding test and enhanced antianxiolytic effect in chronic unpredictable stress paradigm	Mice	Santarelli et al. (2003)
	Anxiolytic and antidepressant‐like behaviour performance (FST & novelty suppressed feeding test)	Rats	Jiang et al. (2005)
Transgenic methods for enhancement	Enhanced neurogenesis but no change in hippocampal‐dependent learning and memory	Mice	Morcuende et al. (2003)
Observational studies	Decreased hippocampal volume in patients with major depressive disorder (positively affected by long‐term treatment with antidepressants)	Human	Malykhin et al. (2010)
	Lower levels of proliferating cells postmortem found in hippocampi of schizophrenic, but not depressed patients	Human	Reif et al. (2006)
	Level of neurogenesis across different mouse strains correlates with learning, but not memory performance (MWM)	Mice	Kempermann and Gage (2002)
Aging			
Enhancement of neurogenesis through learning and/or enrichment	Enhanced learning and memory performance (MWM) & hippocampal‐independent behaviours (locomotion, exploration) Exercise enhanced learning and memory consolidation in MWM	Mice	Kempermann et al. (2002), van Praag, Shubert, Zhao, and Gage (2005)
Observational studies	Impaired learning & memory (MWM & pattern separation) Impaired learning & memory (MWM) in a dose‐dependent manner with reduction in neurogenesis	Rats	Driscoll et al. (2006), Drapeau et al. (2003)
	Impaired learning & memory in MWM due to imprecise adoption of search strategies, correlated with reduced neurogenesis	Mice	Gil‐Mohapel et al. (2013)

## 3 ADULT HIPPOCAMPAL NEUROGENESIS

**Figure 1:**
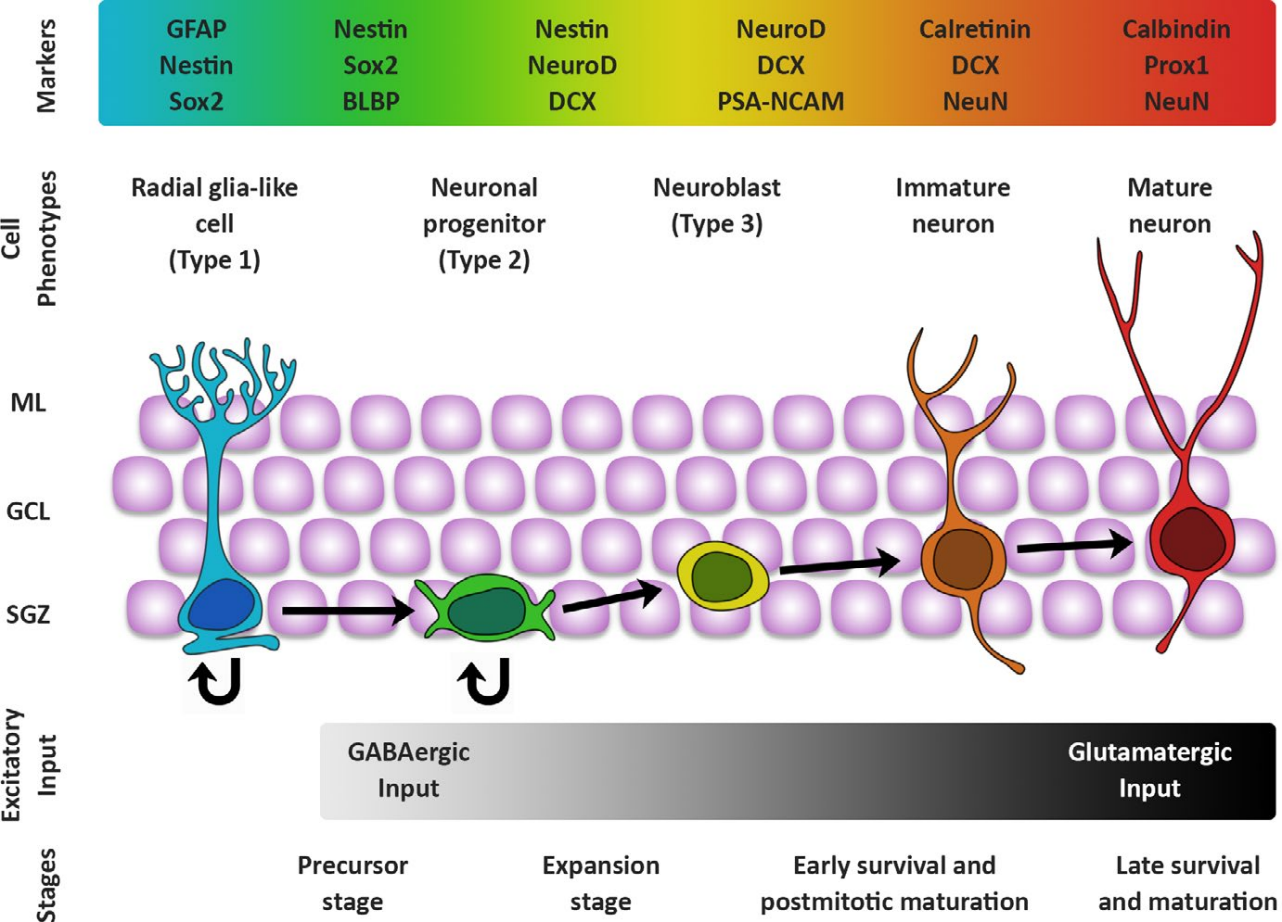
Stages of hippocampal neurogenesis. Depiction of the stages of the neurogenic process in the hippocampus. The radial glia‐like stem cells (Type 1; blue) maintain their pool through self‐renewal and give rise to progenitor cells expressing similar markers but displaying different morphology (Type 2 (A&B); green), which undergo rapid proliferation and begin to express markers specific to the neuronal fate of their progeny. Type 2 cells generate neuroblasts (Type 3; yellow). The neuroblasts enter the early survival stage (orange cells) and extend processes towards the molecular layer. During the late survival stage, only newborn neurons that have formed functional connections and have matured morphologically (red cells) remain from the thousands of neuroblasts generated. Granule neuron somata are represented in purple. The colour‐coded bar on top illustrates the gradual transition in marker expression as the cells progress through the different stages of the neurogenic process. The grey‐gradient‐scale bar on the bottom represents the switch of newborn neurons from GABA to glutamatergic input. ML: molecular layer; GCL: granule cell layer; SGZ: subgranular zone

Adult hippocampal neurogenesis encompasses several consecutive phases of development, which are preserved in the adult brain and result in the production of new neurons: a precursor stage, an early survival stage, a postmitotic maturation stage and a late survival stage (nomenclature adopted from Kempermann, 2015). The stages can be further divided into numerous events or transformations based on evaluating cell morphology and protein expression (Figure 1; Kempermann, 2015; Kempermann, Jessberger, Steiner, & Kronenberg, 2004; Steiner et al., 2006). During the precursor and expansion stages, NSCs go through three continuous progenitor phases characterized by elevated proliferation. These are followed by the early survival stage when NPCs exit the cell cycle and the number of newborn neurons significantly decreases due to elimination. Next, the postmitotic stage is characterized by dendritic and axonal outgrowth, synaptogenesis and the establishment of connections. Finally, the late survival stage marks the integration of new granule cells into the existing circuitry and an increase in synaptic plasticity. It is currently thought that the neuronal maturity process takes around 7 weeks, with a subsequent phase of amplification in synaptic plasticity (reviewed by Kempermann, 2015; Kempermann et al., 2004).

Microglia are also an important regulator of adult hippocampal neurogenesis. Specifically, they phagocytose newborn neurons that fail to integrate into existing circuitry (Sierra et al., 2010); they regulate glutamatergic receptors maturation and synaptic transmission, and support synaptic pruning (Sierra et al., 2014). Additionally, microglia can suppress neurogenesis under inflammatory conditions by exhibiting a neurotoxic phenotype (reviewed by Belarbi & Rosi, 2013), which may have important therapeutic implications for some neurological disorders (reviewed by Eggen, Raj, Hanisch, & Boddeke, 2013).

### 3.1 Human adult hippocampal neurogenesis: Controversies and convergence

More than two decades ago, the rate of proliferation and the process of functional integration of adult‐born neurons into the existing circuitry were reported to be remarkably similar across species (Eriksson et al., 1998). Since then, it has been established that qualitative features of neurogenesis, such as the morphology of newborn neurons, as well as quantitative ones, such as age‐related changes are shared between the murine and human hippocampus (Knoth et al., 2010). A landmark study in 2013 measured the concentration of nuclear bomb test‐derived ^14^C in genomic deoxyribonucleic acid (DNA) and found evidence for the birth of as many as 700 new neurons each day in the adult human hippocampus corresponding to an annual turnover rate of 1.75% (Spalding et al., 2013), which is comparable to that found in middle‐aged rodents. The study also demonstrated that neurogenesis in the human hippocampus was evident in older age, which is in contrast to the age‐related decline previously observed in rodents (Kuhn, Dickinson‐Anson, & Gage, 1996). Since then, discussion in the field has culminated in the proposal that the rate of generation and maturation of newborn neurons are significantly different between rodents and humans (reviewed by Snyder, 2019). This is supported from a study using a model of the cross‐species transformation which has indicated that the developmental span and body size should be taken into account when translating results on hippocampal neurogenesis between rodents and primates (Charvet & Finlay, 2018).

Inconsistent reports over the maintenance of hippocampal neurogenesis over the lifespan in humans have brought about recent debate about its functional significance (reviewed by Lee & Thuret, 2018; Snyder, 2018). In a recent investigation of postmortem brain tissue obtained from 18 adults and 19 perinatal and postnatal samples (age range: 14 gestational weeks to 77 years), it was reported that no newborn neurons were found in the DG of adults and only a few isolated young neurons were observed in samples from young individuals (7–13 years of age). The samples with the most numerous immature neurons observed came from perinatal and postnatal (up to 1 year of age) tissue (Sorrells et al., 2018). On the contrary, using a similar immunohistochemical (IHC) approach, another group of researchers observed immature and mature adult‐born neurons in the hippocampal samples obtained postmortem from 28 healthy individuals (age range: 14–79 years of age) and the number of each cell type was estimated to be at least in the thousands (Boldrini et al., 2018). It is possible that the big discrepancy in results stems from the fact that in the former study, tissue was obtained from individuals suffering a wide range of diseases (although full medical history was not provided), while in the latter study tissue was obtained from healthy individuals (reviewed by Snyder, 2018). Given the similarity in methods employed, both studies clearly demonstrate the limitations and caveats in studying neurogenesis in human postmortem tissue. Importantly, researchers need to produce detailed reports on the medical records of the patients whose tissue has been examined since factors such as postmortem delay and timing of tissue fixation can have a profound effect on protein degradation, specifically in the case of the fast‐degrading doublecortin (DCX), a protein present on immature neurons that has been used as a common marker of neurogenesis (reviewed by Lucassen et al., 2019). The controversy has been somewhat alleviated due to recent research which was carried out under controlled conditions for postmortem tissue processing and timing of fixation, and demonstrated that immature neurons exist in the DG of humans aged up to 90 years (Moreno‐Jiménez et al., 2019). Similarly, Tobin et al. (2019) have demonstrated that hippocampal neurogenesis is persistent through the tenth decade of life. To further address the existence of the phenomenon in the human brain, future studies need to examine not only evidence on an immunohistochemical level but also on a transcriptomic and gene expression level. For instance, single‐cell sorting and sequencing could aid in profiling the cells and establishing whether indeed granule cells born at different stages of the lifespan have unique characteristics and hence should not be considered as one homogenous population (Snyder, 2019). Additionally, an approach for studying hippocampal neurogenesis in vivo has been well characterized but hardly used, namely the use of magnetic resonance spectroscopy where metabolites enriched in stem cells were identified based on their distinct resonance at specific frequency in fatty acids (Manganas et al., 2007). Hence, a combination of technological approaches could aid in advancing our knowledge of the neurogenic process in humans and reconciling the various data obtained from postmortem human tissue.

### 3.2 Function of hippocampal neurogenesis during adulthood

**Figure 2:**
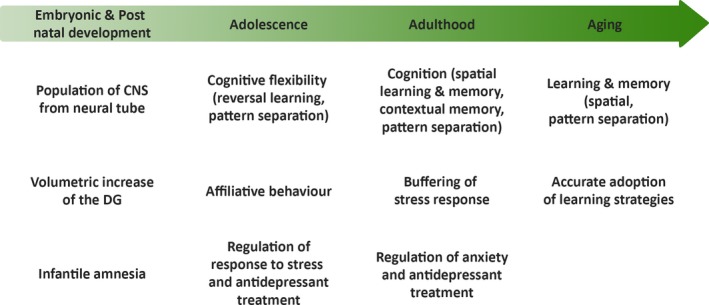
Function of hippocampal neurogenesis. Function of hippocampal neurogenesis through the lifespan as evidenced by literature summarized in Table 1

While some inconsistencies are evident from reports through the years, rodent studies have primarily shown that adult hippocampal neurogenesis is involved in spatial and contextual memory, pattern separation and in mood regulation (Table 1, Figure 2). Studying this causal link has been enabled through the utilization of various ablation techniques such as irradiation, pharmacological interventions (with antimitotic drugs to decrease, or antidepressants to enhance neurogenesis) and transgenic mice (reviewed by Zhao et al., 2008). It should be noted though that some ablation techniques have led to impaired learning performance in certain tasks, which could be due to the complexity of the function of adult‐born neurons and also the fact that the stage of neurogenesis that is targeted by the intervention may influence the specific cognitive task (for a comprehensive review, see Zhao et al., 2008). Furthermore, differences in the specific tests used as well as the species and strains may additionally account for the discrepancies in results. Interestingly, the treatment of brain cancer in humans often requires cranial radiation therapy which has been associated with progressive cognitive decline such as impairments in memory, attention and executive function (Sarkissian, 2005). These side effects have been attributed in part to the decrease in hippocampal neurogenesis that the treatment may cause (Greene‐Schloesser, Moore, & Robbins, 2013). Behavioural studies in rodents where neurogenesis is enhanced through enrichment have shown positive correlation between the level of adult hippocampal neurogenesis and performance on hippocampal‐dependent tasks such as the Morris Water Maze (MWM) and pattern separation (reviewed by Zhao et al., 2008).

Pattern separation refers to the ability to form distinct representations of similar inputs or the process of disambiguating those similar inputs by producing dissimilar outputs (Treves, Tashiro, Witter, & Moser, 2008). This computational process has been suggested to play role in the discrimination between similar memories and to be dependent on the newly generated granule cells in the DG formation (Aimone et al., 2014; Clelland et al., 2009; Deng, Aimone, & Gage, 2010; Snyder, Hong, McDonald, & Wojtowicz, 2005). Despite many findings supporting the involvement of hippocampal neurogenesis in pattern separation (Hvoslef‐Eide & Oomen, 2016), discrepancies in the literature still exist (Cushman et al., 2012; Groves et al., 2013). Interestingly, after performing a systematic review and meta‐analysis of studies using ablation of hippocampal neurogenesis to test its involvement in behavioural pattern separation, and using effect sizes rather than statistical significance (p‐value) as a metric method of evaluating the compatibility between results of different studies, França and colleagues found that the majority of data consistently supported a strong reliance of pattern separation on hippocampal neurogenesis (França, Bitencourt, Maximilla, Barros, & Monserrat, 2017).

As well as being involved in learning and memory, adult neurogenesis has been shown to play a role in mood regulation, and in particular in antidepressant action (Duman, Nakagawa, & Malberg, 2001; Malberg, Eisch, Nestler, & Duman, 2000; Santarelli et al., 2003; Tanti & Belzung, 2013; Warner‐Schmidt & Duman, 2006). In addition, as depression is widely associated with stress‐related disorders, it is worth noting that adult hippocampal neurogenesis also plays a key role in buffering stress responses in animals (Anacker et al., 2018; Petrik, Lagace, & Eisch, 2012; Snyder, Soumier, Brewer, Pickel, & Cameron, 2011). For instance, it has been extensively characterized that exposure to stressors early in life may have profound effects on the different stages of hippocampal neurogenesis and susceptibility to anxiety and depression later in life (reviewed by Fitzsimons et al. (2016, Lucassen et al. (2015, Naninck et al. (2015). Patients suffering from major depressive disorder have presented with reductions in hippocampal volume, which may be reflective of reductions in neurogenesis (Czéh & Lucassen, 2007; Malykhin, Carter, Seres, & Coupland, 2010; Sapolsky, 2000). However, it is worth noting that evidence to position neurogenesis as an aetiological factor in the development of mood disorders is lacking due to the fact that ablation of neurogenesis does not induce depressive‐like or anxiety‐like behaviours in rodents in the absence of another negative stimulus such as a stressor (Petrik et al., 2012; Zhao et al., 2008). It is now two decades since Duman and colleagues showed that antidepressants can increase the number of newborn granule cells in rodent models of depression (Malberg et al., 2000), yet the precise mechanism and function of hippocampal neurogenesis in antidepressant‐mediated mood regulation remain to be elucidated with many controversies arising from different species and methods used by different laboratories (for reviews, see Petrik et al., 2012; Zhao et al., 2008; Table 1).

## 4 HIPPOCAMPAL NEUROGENESIS DURING THE DEVELOPMENTALLY CRITICAL PERIODS OF ADOLESCENCE AND OLDER AGE

### 4.1 Adolescent hippocampal neurogenesis

The levels of neurogenesis in the adolescent rodent hippocampus are much higher compared to adults as illustrated by a mouse study comparing the number of bromodeoxyuridine (BrdU)+ and DCX+ cells between mice at age P30 and mice at age P120. There was a fourfold decrease in the number of proliferating (BrdU+) NPCs and the number of immature neurons (DCX+) from adolescence to adulthood suggesting an adolescent‐associated increase in plasticity coupled with a dramatic reduction in neurogenesis during the transition from adolescence to adulthood (He & Crews, 2007). The mechanisms by or purpose for which this occurs are poorly understood and only a limited number of studies to date have focused on investigating the process of hippocampal neurogenesis during adolescence. Interestingly, it has been shown that the process and time course of apoptosis of newborn granule cells in the adolescent DG closely reflect that of the adult DG, albeit to an exaggerated degree (Curlik, Difeo, & Shors, 2014). Specifically, juvenile rats (P21–P23) were administered an intraperitoneal (i.p.) injection of BrdU and the number of cells, which incorporated BrdU was analysed one and three weeks postinjection. It was found that more than 7,000 proliferating cells retained BrdU expression within a week. However, most of them were no longer detected 3 weeks after the injection, indicating a similar rate of apoptosis as in the adult hippocampus (Curlik et al., 2014; Epp, Spritzer, & Galea, 2007).

#### 4.1.1 Adolescent hippocampal neurogenesis and exercise

Findings from our laboratory have revealed an age‐specific effect of voluntary exercise on hippocampal neurogenesis such that adolescent‐initiated running led to an increased expression of a wide array of plasticity‐ and neurogenesis‐related genes in the hippocampi of Sprague Dawley (*SD*) rats compared to rats that had access to a running wheel during adulthood. Among the upregulated genes were the pro‐neurogenic *bdnf* (brain‐derived neurotrophic factor), *tlx* (nuclear receptor tailless) and *dcx*, and the pre‐ and postsynaptic regulating genes *synaptophysin* and *psd‐95* (postsynaptic density protein 95; O’Leary, Hoban, Cryan, O’Leary, & Nolan, 2019). Moreover, in another cohort of *SD* rats comparing the effects of voluntary exercise initiated either during adolescence or adulthood, we showed that both the number and the complexity (measured by number of neurites, their length and branch points) of DCX+ cells were significantly increased in the hippocampi of rats exercising since adolescence compared to their respective controls. When exercise was initiated during adulthood, hippocampal DCX+ cells presented with increased complexity, but not number, compared to the nonexercising controls (O’Leary et al., 2018).

#### 4.1.2 Adolescent hippocampal neurogenesis and alcohol

Much research has concentrated on investigating the effects of alcohol exposure during adolescence on brain development and cognitive behaviour (reviewed by Crews, Vetreno, Broadwater, and Robinson (2016). Interestingly, it has been consistently shown that hippocampal neurogenesis is significantly reduced in adolescent and adult rodent models of binge drinking. However, while neurogenesis recovers to normal levels in adults after a period of abstinence, when the alcohol exposure took place during adolescence rather than adulthood the deficit persists until late adulthood (Crews, He, & Hodge, 2007). Such a long‐lasting reduction in hippocampal neurogenesis was also observed after administration of alcohol to adolescent rhesus monkeys (Taffe et al., 2010). Furthermore, binge ethanol exposure during adolescence has been shown to directly influence NSCs and NPCs but not neuroblasts, by reducing proliferation and increasing apoptosis in cells of both the dDG and the vDG of rodents (rats; Vetreno & Crews, 2015) and nonhuman primates (rhesus monkeys; Taffe et al., 2010). This was also coupled with impaired cognitive performance on memory tests (Taffe et al., 2010; Vetreno & Crews, 2015). Moreover, during the abstinence period in adolescent rodents, neuroblasts were ectopic and were found in the molecular layer of the DG rather than within the SGZ (McClain, Morris, Marshall, & Nixon, 2014). Collectively, these studies not only highlight the sensitivity of the adolescent brain to positive environmental factors such as voluntary exercise and negative ones such as alcohol exposure, but also emphasize that altered hippocampal neurogenesis during adolescence may be an important factor which underpins susceptibility to changes in hippocampal‐dependent cognitive function in later life. Further research will delineate the functional relevance of hippocampal neurogenesis to environmental influences during adolescence.

### 4.2 Function of hippocampal neurogenesis during adolescence

#### 4.2.1 Studies inhibiting neurogenesis

Several studies have investigated whether inhibiting hippocampal neurogenesis during adolescence results in similar impairments as observed when the process was inhibited in adulthood (Table 1, Figure 2). For instance, cranial irradiation of the rat hippocampal region during preadolescence resulted in a dramatic increase in apoptosis and impaired production and release of growth factors in the hippocampus, while the same procedure performed in adulthood resulted in sustained release of pro‐inflammatory cytokines in the hippocampus (Blomstrand, Kalm, Grandér, Björk‐Eriksson, & Blomgren, 2014). Chronic stress induced a transient reduction in the number of proliferating NPCs in the hippocampi of adolescent but not adult male mice suggesting a resilience by adolescent mice to impairments induced by the stress. This phenomenon could not be accounted for by the damage to emotional processing and sociability caused by the inhibition of hippocampal neurogenesis since both adolescents and adults exhibited normal performance on depression‐related behavioural tests as well as a regular corticosterone response after acute exposure to stress (Kirshenbaum, Lieberman, Briner, Leonardo, & Dranovsky, 2014). Interestingly, using the same method of transient ablation of hippocampal neurogenesis, another group of researchers found the same outcome of memory and anxiety‐related behaviours for both adolescent and adult female mice. However, impaired female–female social interaction resulted when neurogenesis was inhibited during adolescence but not adulthood, reflected by a complete social aversion towards conspecifics, as well as impaired pup retrieval (Wei, Meaney, Duman, & Kaffman, 2011). We have also shown that social isolation stress during adolescence can lead to impaired exercise‐induced increased neurogenesis in the hippocampus (Kozareva, O’Leary, Cryan, & Nolan, 2018). Interestingly, early life stress exposure during the postnatal period (from P2 to P9) also blunts the pro‐neurogenic effects of exercise when examined at the end of adolescence and start of adulthood (P56) (Abbink, Naninck, Lucassen, & Korosi, 2017).

#### 4.2.2 Studies enhancing neurogenesis

When the effect of the antidepressant fluoxetine administered to rats during either adolescence and adulthood on hippocampal neurogenesis and serotonin synthesis was compared, it was shown that treatment with fluoxetine during adolescence but not adulthood increased neurogenesis and serotonin synthesis in the vDG but not dDG (Klomp, Václavů, Meerhoff, Reneman, & Lucassen, 2014). Additionally, adolescent‐ versus adult‐initiated voluntary exercise in rats had differential effects on performance on cued‐ and context‐dependent fear conditioning with adult‐initiated exercise enhancing performance on both tasks without influencing the expression of neurogenic and plasticity markers, while adolescence‐initiated exercise did not change performance on the fear conditioning tasks but enhanced expression of neurogenesis and plasticity markers (O’Leary et al., 2019). Our research has further demonstrated that adolescent‐ but not adult‐initiated exercise in rats was associated with an increase (albeit transient) in performance on pattern separation in touchscreen‐based task coupled with increased neurogenesis (O’Leary et al., 2018). Interestingly, a stronger positive correlation between the neurite length of new neurons and cognitive flexibility as measured by reversal learning on a touchscreen‐based task was observed in response to the adolescent compared to adult‐initiated exercise (O’Leary et al., 2018). Moreover, a greater degree of complexity in the new neurons in the hippocampus of rats exposed to exercise during adolescence compared to adulthood was reported (O’Leary et al., 2018).

#### 4.2.3 Summary and perspective from human studies

Such comparisons of treatment and ablation outcomes between adolescence and adulthood, however, need to be considered in the context of not only differences in the basal levels of neurogenesis across development, but also in terms of the hormonal and behavioural changes that occur during the adolescent period. Though limited in number, rodent studies have consistently shown that ablation of neurogenesis during adolescence results in decreased proliferation and survival of hippocampal NPCs from adolescence until late in adulthood, which also correlates with an impairment in performance on memory‐related tasks such as fear conditioning and the MWM test (Achanta, Fuss, & Martinez, 2009; Rola et al., 2004). Similarly, radiation therapy for children and adolescents with cancer is associated with lasting changes in intelligence quotient (IQ) scores and cognitive performance (Rodgers, Trevino, Zawaski, Gaber, & Leasure, 2013). Defective hippocampal neurogenesis during adolescence has been suggested as a contributing factor to the onset and development of neuropsychiatric disorders (reviewed by Hueston, Cryan, & Nolan, 2017), which in combination with the fact that adolescence is a period of dramatic vulnerability to the effect of extrinsic influences, means that it is imperative to expand our understanding of how positive and negative regulators of hippocampal neurogenesis such as stress and exercise influence the brain during this critical period.

### 4.3 Hippocampal neurogenesis during aging

#### 4.3.1 Age‐related decline in neurogenesis across species

Hippocampal neurogenesis is presumed to persist throughout the lifespan; however, a decline in neurogenesis has been recognized to occur with age across species. In fact, Altman and Das (1965) in their pioneering paper commented on the decrease in cell birth within months after birth (Altman & Das, 1965; Kempermann, 2015; Klempin & Kempermann, 2007). The first report to quantify age‐related changes in adult hippocampal neurogenesis came from a study of 12‐ to 21‐month old rats where the authors showed, through BrdU labelling and IHC analysis that a decrease in mitotic activity of NPCs in the SGZ occurred and was associated with a net decrease in neurogenesis (Kuhn et al., 1996). Furthermore, Kempermann and colleagues showed a similar age‐related decrease in hippocampal neurogenesis in 8‐ to 20‐month old mice, and that the decrease in neuronal survival could be somewhat ameliorated by enriched housing conditions (Kempermann, Kuhn, & Gage, 1998).

The age‐associated decrease in hippocampal neurogenesis has also been shown in tree shrews. This study further demonstrated that older animals were more susceptible to a stress‐induced decline in NPC proliferation than their younger counterparts (Simon, Czéh, & Fuchs, 2005). Interestingly, despite a net decrease in hippocampal neurogenesis in wild‐living aged squirrels and chipmunks, it was shown that there was a species difference in terms of the age‐related decrease observed. Specifically, the number of proliferating NPCs was decreased in the DG of squirrels, while the number of immature adult‐born neurons was diminished in the DG of chipmunks (Barker, Wojtowicz, & Boonstra, 2005). This finding is particularly interesting, in light of the complexity of studying the neurogenic process in noncaptive populations, since the squirrels were relying on neurogenesis‐dependant strategies (spatial memory) to locate their hidden food stores, while the chipmunks had much less developed spatial memory and relied on a single place for food (Barker et al., 2005).

Aside from rodents, it has been shown using BrdU incorporation that hippocampal neurogenesis persists in nonhuman primates, namely the Macaque monkeys, until they are 23 years old (the human equivalent of old age). However, the rate of neurogenesis occurred at significantly lower levels than during adolescence and adulthood (Gould, Reeves, et al., 1999). Similar to what has been reported for adult human hippocampal neurogenesis, this existence of an age‐related decline in neurogenesis remains controversial (Kempermann et al., 2018). Intriguingly, the researchers who propose the occurrence of neurogenesis in the adult human hippocampus have not found a decline of NPC proliferation or of neurogenesis with age (Boldrini et al., 2018; Eriksson et al., 1998), despite a reported age‐associated decline of the quiescent progenitors pool (Boldrini et al., 2018). A recent study of a cohort of 18 participants with mean age 90.6 years demonstrated that both NPCs and neuroblasts were identified in the hippocampi albeit with high between‐subject variation. Interestingly, cognitive status was positively correlated with the number of newborn neurons identified in these subjects (Tobin et al., 2019). Similarly, Moreno‐Jiménez et al. (2019) showed a negative correlation between the number of immature neurons and age in neurologically healthy humans, but that the number of new neurons dropped sharply in patients with AD. The authors suggest that impaired hippocampal neurogenesis may be a mechanism underlying the memory deficits in AD, which opens up further questions on the mechanistic role of hippocampal neurogenesis in cognitive function across the lifespan.

#### 4.3.2 Possible mechanisms for age‐related decline in hippocampal neurogenesis

The mechanisms underlying the age‐related decline in hippocampal neurogenesis remain poorly understood. It has been proposed that within the senescent brain the neurogenic niche may be deprived of the extrinsic signals regulating the neurogenic process or that the aged NPCs are less responsive to normal signalling within the niche, or both (Kempermann, 2015). The evidence accumulated thus far points to changes in the properties of the neurogenic niche with age, rather than changes in the phenotype of the NS/PCs themselves. For instance, it has been reported that the numbers of NSCs and NPCs as well as the proportion of astrocytes to neurons in the hippocampus of young and aged rats remained the same; however, there was a decrease in the number of cells actively undergoing mitosis in the aged animals (Hattiangady & Shetty, 2008). The authors speculated that this was due to changes in the milieu of the neurogenic niche based on their earlier observations that important regulators of neurogenesis such as BDNF and CREB (cyclic adenosine monophosphate (cAMP)‐response element binding protein) decreased dramatically in the DG of middle‐aged and aged rats (Hattiangady, Rao, Shetty, & Shetty, 2005). Additionally, it was shown that the gradual loss of hippocampal neurogenesis in aged mice was associated with downregulation of the mitotic factor survivin in a Wnt‐dependent signalling manner (Miranda et al., 2012). This finding was corroborated with the observation that the Wnt antagonist Dickkopf‐1 increased with age, while mice deficient in Dickkopf‐1 not only exhibited enhanced hippocampal neurogenesis during aging, but also performed better at neurogenesis‐dependent tasks, involving spatial working memory, than age‐matched controls whose Dickkopf‐1 expression was not modulated (Seib et al., 2013).

Growth factors such as epidermal growth factor (EGF) and insulin‐like growth factor 1 (IGF‐1), which are important regulators of adult neurogenesis, have also been shown to play a role in the age‐related decline of hippocampal neurogenesis in the rodent (reviewed by Kempermann, 2015). For instance, intracerebroventricular (i.c.v.) administration of basic fibroblast growth factor (FGF‐2) and EGF resulted in not only a reversal in age‐related decrease in neurogenesis, but also an enhancement of the number of adult‐born neurons in the aged hippocampus, illustrating that the aged brain is still susceptible to the influence of exogenous growth factors (Jin et al., 2003). Moreover, the levels of FGF‐2 as well as IGF‐1 and vascular endothelial growth factor (VEGF) were found to dramatically decline in the hippocampi of aged rats (Shetty, Hattiangady, & Shetty, 2005). Additionally, this FGF‐2 decline was as a result of an age‐related deterioration of FGF‐2 synthesis by astrocytes, leading to a reduced number of glial fibrillary acidic protein (GFAP)+FGF2+ radial glia‐like cells in the DG of aged rats (Shetty et al., 2005). It was also independently demonstrated that the hippocampus is one of the regions of the rat brain with the highest and most robust expression of the FGF‐2 receptor FGFR2, specifically on astrocytes, and that the expression of this protein decreased significantly with age (Chadashvili & Peterson, 2006). Infusion of IGF‐1 through i.c.v. ameliorated the decrease in hippocampal neurogenesis in aged rats (Lichtenwalner et al., 2001), while in a model of long‐lived mice (the Ames dwarf mice) enhanced hippocampal neurogenesis coupled with increased levels of IGF‐1 was observed during aging (Sun, Evans, Hsieh, Panici, & Bartke, 2005).

A prominent perpetrator of the age‐related decline in hippocampal neurogenesis has been proposed to be the family of glucocorticoid hormones and receptors, the release and circulation of which coincidentally increase with age (Cameron & McKay, 1999). Glucocorticoids have been linked to increased hippocampal atrophy and to regulate adult hippocampal neurogenesis (Egeland, Zunszain, & Pariante, 2015; Odaka, Adachi, & Numakawa, 2017; Sapolsky, 2000). Another piece of evidence supporting the impairment in the neurogenic niche properties over time stems from a study where aged mice were infused with vascular and neurogenic factors of young mice which resulted in a rejuvenated neurogenic niche and a restoration of hippocampal neurogenesis (Katsimpardi et al., 2014). Furthermore, despite the lack of alterations in properties of hippocampal NSCs with age, a possible delay in the maturation of adult‐born neurons in the aged DG has been demonstrated (Rao, Hattiangady, Abdel‐Rahman, Stanley, & Shetty, 2005).

As regulators of neurogenesis during embryonic and adult neurogenesis, microglia have also been proposed as significant modulators of neurogenesis in the aging brain. The first line of evidence came from studies showing that CD200 and the fractalkine (CX3CL1), which are key regulators of the microglia‐neuronal crosstalk, were disrupted in the aged brain. This could play a role in the increased microglia activation and reduced hippocampal neurogenesis that occur with age (for review see Gemma, Bachstetter, & Bickford, 2010). Interestingly, when primary microglia cultures prepared from mice that had undergone exercise (to prime a pro‐neurogenic microglial phenotype) were added to hippocampal preparations of aged mice, an activation of latent NPCs was observed (Vukovic, Colditz, Blackmore, Ruitenberg, & Bartlett, 2012). Minocycline‐induced blockade of microglia activity in adult and aged mice resulted in improved performance in the MWM task, a reduced number of activated microglia in aged but not adult mice and increased neurogenesis in the adult, but not aged mice (Kohman, Bhattacharya, Kilby, Bucko, & Rhodes, 2013). However, under pathological aging conditions, using a murine model of the neurodegenerative prion disease, it was found that stimulation of microglia proliferation corresponded to increased neurogenesis, while inhibiting microglia proliferation resulted in decreased neurogenesis (De Lucia et al., 2016). Our knowledge of the involvement of microglia in hippocampal neurogenesis in the human aged brain is limited due to current limitations in human postmortem tissue processing methods as outlined previously. However, it has been proposed that understanding the mechanisms by which microglia regulate hippocampal neurogenesis may contribute to the development of intervention strategies for reducing the burden of age‐related diseases such as stroke and AD (reviewed by Koellhoffer, McCullough, and Ritzel (2017). Taken together, these findings highlight the complex interplay of different factors within the neurogenic niche that may be affected by the aging process and which thereby ultimately affect the number of immature neurons produced in the aged brain.

#### 4.3.3 Function of hippocampal neurogenesis during aging

Adult hippocampal neurogenesis has been proposed to be a key element in ensuring and maintaining functional hippocampal integrity in old age (Kempermann, 2015; Kempermann, Gast, & Gage, 2002). Neurodegenerative diseases due to the age‐dependent rapid and continuous loss of neurons (such as Parkinson's and Huntington's disease) have been suggested to reflect the contraposition of the neurogenic process such that under homoeostatic conditions a fine balance between neurodegeneration and neuroregeneration exists, and under pathological conditions, the balance is disturbed and a disease manifests (Kempermann, 2015). Even though little evidence has accumulated in support of this theory, if it proves correct, it in combination with findings regarding the high potential of stem‐cell‐based strategies for the treatment of age‐related neurodegenerative disorders, make the hypothesis that adult neurogenesis holds a key to novel therapeutic approaches in the treatment of age‐related neurodegenerative disorders rather attractive (Lindvall & Kokaia, 2015; Lindvall, Kokaia, & Martinez‐Serrano, 2004).

Decreased hippocampal neurogenesis is proposed as an important mechanism underlying age‐related cognitive decline as well as neurodegenerative disorders such as AD and various types of dementia (Kuzumaki et al., 2010). Evidence in this regard was recently published in two separate recent studies examining hippocampal neurogenesis in human tissue from people suffering mild cognitive impairment and AD. Both studies demonstrated a dramatic decrease in the number of NPCs and neuroblasts in hippocampal tissue from AD patients which was related to the stage of the disease (Moreno‐Jiménez et al., 2019; Tobin et al., 2019). Interestingly, a decrease in the number of newborn neurons was observed in AD patients at the very early stage of the disease when the characteristic neurofibrillary tangles and senile plaques had not become prevalent (Moreno‐Jiménez et al., 2019). This suggests a potential for using neurogenesis levels as an early biomarker of the disease. While Moreno‐Jiménez et al. (2019) found a correlation between the stage of AD and the rate of decrease in neurogenesis, no correlation was apparent in the cohort of subjects examined by Tobin et al. (2019). In both instances, however, there was a large between‐subject variation in number of newborn neurons. Thus, while these studies have opened up significant avenues of research on the topic, larger cohorts need to be employed to better understand the hippocampal neurogenesis in age‐related neurodegenerative disorders. Furthermore, the mechanisms of how hippocampal neurogenesis could possibly function as therapeutic target for neurodegenerative conditions remain to be examined.

Similar to studies on adult hippocampal neurogenesis, the function of hippocampal neurogenesis in rodents during aging has been studied using neurogenesis enhancing approaches in conjunction with neurogenesis‐associated cognitive tasks in aged animals under normal physiological conditions (Table 1, Figure 2). Given the positive correlation between physical activity and the reduced risk of dementia and cognitive decline in an elderly cohort (Laurin, Verreault, Lindsay, MacPherson, & Rockwood, 2001), the Gage laboratory investigated whether hippocampal neuroplasticity may account for the cellular mechanism underpinning these observed benefits using a rodent study approach. To test their hypothesis, the authors exposed middle‐aged mice (10 months old) to an enriched environment, consisting of a rearrangeable set of plastic tubes, a running wheel, and nesting materials and toys for the duration of the 10‐month study, a period in mice considered to reflect senescence in humans (Kempermann et al., 2002). Interestingly, the mice exposed to the enriched environment displayed a fivefold increase in the number of newborn neurons compared to controls, which was coupled with significant enhancements of their learning and memory performance on the MWM task, as well as exploratory behaviour in an open field task and locomotor activity on the rotarod. This suggests that living in a stimulating environment during aging can induce an increase in hippocampal neuroplasticity and cognitive performance (Kempermann et al., 2002).

Researchers employing neurogenesis‐associated behavioural tests, which probed spatial memory and pattern separation in aged rats, found a positive correlation between structural alterations and neurogenesis in the hippocampus, and performance on the behavioural tests (Driscoll et al., 2006). Specifically, with the advancement of age, rats displayed decreased hippocampal volume and hippocampal neurogenesis, which was paralleled by impairments in cognitive performance on the MWM task and a pattern separation paradigm (Driscoll et al., 2006). More recently, a study in mice examined the effects of senescence on the different stages of hippocampal neurogenesis on both learning and spatial memory performance on the MWM task. The results illustrated that the decline in neurogenesis over time could best be modelled by an exponential inverted U‐shape curve, such that the most rapid decline occurred between 3 and 6 months of age, after which neurogenic levels slowly but steadily decreased. Interestingly, the decrease could be accounted for in all stages of the neurogenic process, namely proliferation, differentiation and survival. What is more, the authors found that performance in the MWM task was progressively worse with age not due to impairments in learning, but due to mice adopting more spatially imprecise strategies over time (Gil‐Mohapel et al., 2013).

As well as decreased neurogenesis, neurodegenerative diseases are also characterized by neuronal loss primarily due to apoptosis (Lunn, Sakowski, Hur, & Feldman, 2011). Upon examination of the effect of genetically induced hippocampal neuronal loss, in the aging mouse brain, it was found that loss of neurons in the CA1 and DG areas enhanced hippocampal neurogenesis. However, despite the increase in neurogenesis, these transgenic animals performed significantly poorer on spatial memory in the Barnes maze. The results thus indicated that the increase in the number of granule cells did not mitigate the cognitive deficit observed with aging (Yeung et al., 2014). Thus, even if neurogenesis is stimulated as a result of endogenous cell loss, the increased numbers of newborn neurons cannot rescue the apoptosis‐associated cognitive impairment. This implies that neurogenesis at specific time points of the lifespan may have different functions. A simpler alternative explanation would be that rather than distinct functions, the rate of neurogenesis that changes across the lifespan may account for the different resultant cell phenotypes observed, although this question remains to be addressed. Thus, the current consensus is that neurogenesis alone cannot account for the age‐related cognitive decline observed in rodents and humans, and more mechanisms need to be taken into account for successful development of preventative and therapeutic strategies to ameliorate the deterioration of cognitive function during senescence (Kempermann, 2015).

## 5 CONCLUSION

The field of hippocampal neurogenesis has become firmly established over the last decades, and research of this topic has significantly expanded our knowledge and understanding of the properties of NSCs, the stages of the neurogenic process and the potential functional roles of newly generated granule cells. The impact on our perspective of brain plasticity and its potential under pathological and physiological conditions has fostered current studies to continuously examine and manipulate NSCs to further explore their regenerative capacity. However, significant challenges still remain. Firstly, the evidence accumulated across species needs to be reconciled in terms of the differences in rate at which neurogenesis occurs, the different stages of the lifespan at which neurogenesis peaks and the functional consequences of such species‐specific variations. The question as to whether hippocampal neurogenesis adopts different functions at different times across the lifespan or whether the rate of neurogenesis across life can explain the differences in functions remains to be explored. Further, the approach for studying neurogenesis in humans needs to be adapted to meet the challenges presented by working with human samples. The heterogeneity of the neurogenic niche needs to be acknowledged and further evaluated using techniques such as single‐cell sequencing that would help us better define the properties of the cells during different stages of the process as well as the changes that newly born granule cells undergo between birth and full maturation. Lastly, reconciling the role of neurogenesis in hippocampal function remains to be elucidated. For this, studies need to employ more selective, inducible and reversible manipulations of the neurogenic process in vivo. To conclude, this review highlights autonomy of hippocampal neurogenesis across adolescence, adulthood and aging in different species. Neurogenesis in the adult rodent brain has already been extensively characterized. Now, using this knowledge in conjunction with new technologies will bring us closer to understanding the process of hippocampal neurogenesis across the lifespan in rodents as well as humans and to assimilating ways in which it can be used for improving brain health.

## CONFLICT OF INTEREST

None declared.

## AUTHOR CONTRIBUTIONS

D.A.K. and Y.M.N. wrote the paper. J.F.C. assisted with the format of the manuscript.
